# A study of cross-border E-commerce research trends: Based on knowledge mapping and literature analysis

**DOI:** 10.3389/fpsyg.2022.1009216

**Published:** 2022-12-14

**Authors:** Yongfeng Chen, Mengya Li, Jiajie Song, Xueli Ma, Yiding Jiang, Sainan Wu, Guan Lin Chen

**Affiliations:** ^1^College of Science and Technology, Ningbo University, Cixi, Zhejiang, China; ^2^College of Computer Science and Technology, Zhejiang University of Technology, Hangzhou, China; ^3^Department of Distribution Management, Shu-Te University, Kaohsiung, Taiwan

**Keywords:** cross-border e-commerce, knowledge graph, mixed research, trend of development, bibliometric analysis

## Abstract

As a result of the trend toward economic globalization, the vigorous development of cross-border e-commerce has attracted many scholars to study this field, involving many related fields, such as consumer behavior, advertising, information systems, and supply chain management. Throughout the existing literature, it can be found that most of the research focuses on certain influencing factors of the development of cross-border e-commerce, and there is no systematic and macro- overview of the development trend of research in this field in recent years, nor the integration and analysis of keywords. Given that the research in the field of cross-border e-commerce is fragmented to a large extent, to effectively explore the research trend in this field, we must understand the current situation of cross-border e-commerce. Systematic bibliometric analysis can solve this problem by providing publishing trends and information on various topics. Therefore, based on the scientific database web, this study collected 198 references related to cross-border e-commerce from 2016 to 2021, briefly introduced the current situation and development trend of cross-border e-commerce, visually analyzed and refined the journals, authors, research institutions, countries, publication years, keywords, citations of academic publications in this field, and other key information, and summarized the development trend and path of CEBC in the existing research. It is helpful for researchers to solve the current research gap, understand the future research direction in this field, and help academia establish a strict knowledge system.

## Introduction

With the continuous development of Internet technologies in recent years, trade between countries has grown closer, and economic development has gradually moved toward globalization and integration. Cross-border e-commerce has great potential for development in countries or regions with similar geographical and cultural characteristics. In other words, it has the potential to develop new revenue models or methods both domestically and internationally (Cho and Lee, 2017). The infinite possibilities of cross-border e-commerce have prompted it to enter a new stage of rapid development. Cross-border e-commerce has not only successfully broken traditional trade barriers between countries but also promoted world trade gradually by encouraging merchants and consumers to participate in inter-enterprise trade (global B2B) and transactions between consumers and enterprises (global B2C). The move toward borderless trade will also bring a series of major changes affecting economies and trade patterns worldwide. According to research by Cho and Lee (2017), one-third of e-commerce represents cross-border e-commerce. Taking the European Union (EU) as an example, 15% of overseas sellers offered products to consumers in the EU through e-commerce channels in 2014 (Kawa and Zdrenka, 2016), a 25% increase from previous years (Kawa and Zdrenka, 2016). The development history of cross-border e-commerce shows that it will provide huge space and opportunities for global economic growth in the near future (Wei et al., 2019). In recent years, research in the area of cross-border e-commerce has received more attention as a result of the industry’s rapid growth. Deng and Wang (2016) found that the pioneers of third-party platforms relying on cross-border e-commerce have advantages over the latter in terms of learning effects and conversion costs through online search and mining methods, and they can better solve the cost, technology, and market inconsistencies. Liu and Lai (2016) further summarizes and discusses the current status of the cross-border third-party logistics market, transportation business models, logistics service applications, and the impact of integration with my country’s cross-border e-commerce on the development of cross-border e-commerce. Valarezo et al. (2018) explore the determinants of an individual’s decision to implement cross-border e-commerce. Zhang et al. (2019) analyzed the characteristics and influencing factors of talent demand and concluded that there is a large gap in the demand for cross-border e-commerce talents, while Cheng et al. (2019) created a design based on the four capabilities of market knowledge, technical skills, analytical ability, and business practice ability. A cross-border e-commerce talent training model was developed to address this gap, and the effectiveness of the model was evaluated. Gao (2021) studied the influence mechanism of the application of blockchain technology in cross-border e-commerce on consumers’ purchase intention, explained the application status of blockchain technology in various fields of cross-border e-commerce, and based on this, divides the quality of the cross-border e-commerce blockchain system into three dimensions: commodity information quality, logistics service quality, and payment security.

A review of previous research reveals that the majority of articles on cross-border e-commerce concentrate on a particular aspect that has influenced the industry’s growth rather than providing a comprehensive description of the industry’s development trend in recent years. The lack of integrated analysis of keywords in the cross-border e-commerce field and the lack of statistical analysis on specific time points. With the development of globalization, there are many research branches in the field of cross-border e-commerce, and there are many factors affecting its development. Therefore, systematically summarizing the development trend of cross-border e-commerce has important reference significance for promoting its development and for researchers entering the field of cross-border e-commerce research. This research mainly uses Citespace as a tool for bibliometric analysis and selects SSCI and SCI journals included in WOS from 2016 to 2021 for analysis. This study analyzes key information such as journals, authors, research institutions and countries, publication years, keywords, and citations of publications to establish a knowledge map of cross-border e-commerce research in order to understand the basic characteristics and dynamic changes of cross-border e-commerce. The analysis provides certain theoretical guidance for follow-up research on the development of cross-border e-commerce.

This research will adopt the methods of bibliometrics and content analysis and use CiteSpace software to analyze the research process and future development trends in this field, hoping to solve the following problems: (1) Understand the fundamental characteristics of the literature in the field of cross-border e-commerce from 2016 to 2021. (2) Using the number of papers published as an indicator, create a collaboration network from three perspectives: authors, institutions, and countries to investigate the research status of cross-border e-commerce. (3) Create a keyword co-occurrence map and analyze keyword clusters to understand the overall changing trend of the cross-border e-commerce field. (4) Analyze the current situation of cross-border e-commerce development through content analysis.

## Literature review

### Cross-border e-commerce

Cross-border e-commerce (CBEC) refers to cross-border logistics transactions between multiple parties from different customs regions through e-commerce platforms (Ai et al., 2016). Typical participants are the two main players (buyers and sellers), e-commerce platforms (cross-border online platforms), and other third-party service companies (cross-border logistics providers and payment providers). International buyers order products through online e-commerce platforms, and cross-border transactions are handled by third parties (such as logistics companies or payment companies; Mou et al., 2019a). With the development of electronic information technology and the deepening of economic globalization, great changes have taken place in the consumption patterns and demands of consumers. Supported by growing demand and favorable policies, cross-border e-commerce is developing vigorously in the global environment and has become an important channel for promoting international trade (Li and Chan, 2016; Kim et al., 2017). Cross-border e-commerce, the process of selling goods directly to foreign consumers through digital intermediaries, has received increasing attention over the past few decades (Sinkovics et al., 2007; Giuffrida et al., 2020). By 2022, business-to-consumer (B2C) cross-border online sales are expected to account for 22% of total global e-commerce (Forrester Research, 2019). Based on its economic surplus and rapid growth, there is a broad consensus that CBEC has become one of the most important pillars of international trade (UNCTAD, 2016).

However, cross-border e-commerce products have long transportation times, high-quality return services are difficult to achieve, and transportation costs are too high. Ding et al. (2017) pointed out that the development of cross-border e-commerce will continue to face obstacles such as cultural differences between countries, consumer behavior, laws and regulations, product and marketing issues, payment conditions, and logistics restrictions. Strzelecki (2019) studied the characteristics required to accurately identify customer needs when e-tailers provide services to customers. The willingness to repurchase can reflect the subjective probability of consumers buying from the same store repeatedly (Wu et al., 2014). (Mou et al. 2019b) studied the relationship between customer repurchase intention and actual purchase behavior in the future. Strong repurchase intentions can attract more buyers for the company and increase the market share of the store or enterprise. Although electronic services are gaining popularity around the world, there are still strong uncertainties. Therefore, trust in a certain enterprise or commodity occupies an important position in the minds of customers and is an important determinant of their acceptance of electronic services (Mou et al., 2016).

In the past, there have been many discussions on the positive impact of cross-border e-commerce on the economy and its potential growth and future development; challenges and opportunities for both the supply and demand sides of the market; intensified price competition; improved retail efficiency; positive impact on production in other industries; impact on individuals; and a study of household consumer benefits, labor productivity, and GDP growth. In addition, research on cross-border e-commerce suppliers and consumers has gradually increased. For example, by analyzing the online shopping situation that determines consumers’ purchase intentions, four kinds of clues that promote this kind of consumer behavior can be identified, namely online promotion clues; content marketing clues, personalized recommendation clues, and social comment clues. Additionally, brand familiarity is introduced into the analysis of the influence of cross-border online shopping on consumers’ purchase intention. It is concluded that these four contextual cues for cross-border online shopping have a significant positive impact on consumers’ purchase intentions (Xiao et al., 2019). There is no doubt that the choice of partnership between enterprises is an important factor affecting cross-border e-commerce. On the basis of a literature review, Huang et al. (2021) concluded that the good reputation of enterprises, trust between enterprises, and information sharing are all conducive to the realization of cooperation, thus constructing a theoretical model of partner selection for cross-border e-commerce enterprises. An in-depth discussion on the choice of partners for cross-border e-commerce enterprises under the B2B model. According to relevant reviews, previous studies have not explored the relevant literature process of cross-border e-commerce. Therefore, this study summarizes the context of cross-border e-commerce research through bibliometric methods.

## Research methodology

The Web of Science (WOS) is an online multidisciplinary literature database in which current research is reviewed based on high-quality journal articles in order to obtain valuable information. This study conducted bibliometric data analysis since 2016 mainly in that bibliometric methods mostly adopt a time period as the scope of search. For example, a time period of more than 5 years can be regarded as a scope of search. In order to ensure the rationality of data collection and reduce the deviations and errors in time selection (Xu et al., 2022); the time period from 2016 to 2021 was considered as the scope of search for this study. The reasons why the WOS database was used include: First, this database contains the main literature from SCI and SSCI journals. Second, it is viewed as a source of literature from core journals by most countries and researchers. Lastly, other databases, such as SCOPUS and Science Direct, were not employed in this study because most of them collect papers from seminars or non-English papers, which can lead to deviations and errors in data analysis (Su et al., 2020; Jia et al., 2022). Based on the above reasons, the WOS database was adopted in this study. In terms of data retrieval, the main research is mainly searched through the keyword “Cross Border e-commerce” or “Cross Border Electronic Commerce” or “Cross-border e-commerce” in the WOS database, and conducted according to the time point from 2016 to 2021. In the end, a total of 223 articles were obtained. After selecting the “article” type to search SCI and SSCI journal articles, 23 articles were deleted, and two articles were deleted according to the subject and abstract. The exclusion factors included irrelevant cross-border e-commerce, literature and trend analysis, non-empirical research, and non-English studies (Su et al., 2020; Lin et al., 2022; Zhu et al., 2022). Finally, the contents of 198 articles were retained, and a bibliometric mapping analysis was obtained.

Furthermore, in order to effectively carry out data analysis, this study referred to Nagariya et al. (2021) and Lin et al. (2022) in terms of clustering analysis. Content categories were supplemented objectively through multiple methods to reduce the deviations, errors, and shortcomings of graphic results of conventional bibliometric methods. With respect to content analytical methods, 198 papers were systematically processed. Specifically, two university professors and three researchers read the papers and then double-checked them to ensure content correction.

**Figure 1:**
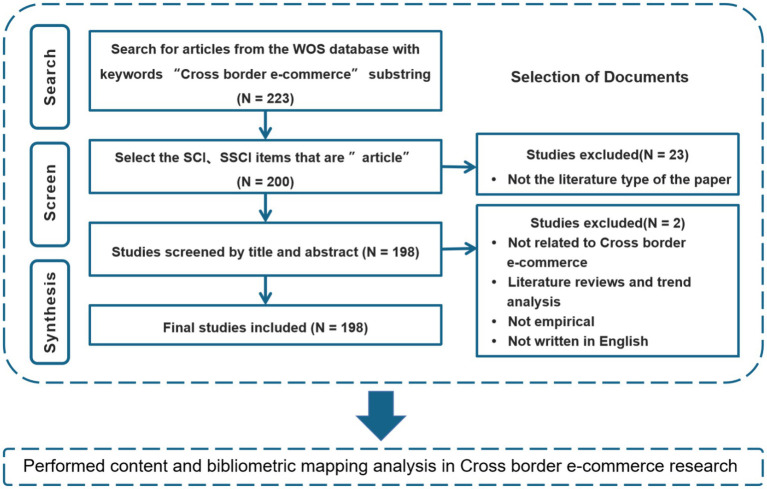
Procedure framework.

The procedure framework of this research is as follows: Figure 1.

## Results

### Publication trends

**Figure 2:**
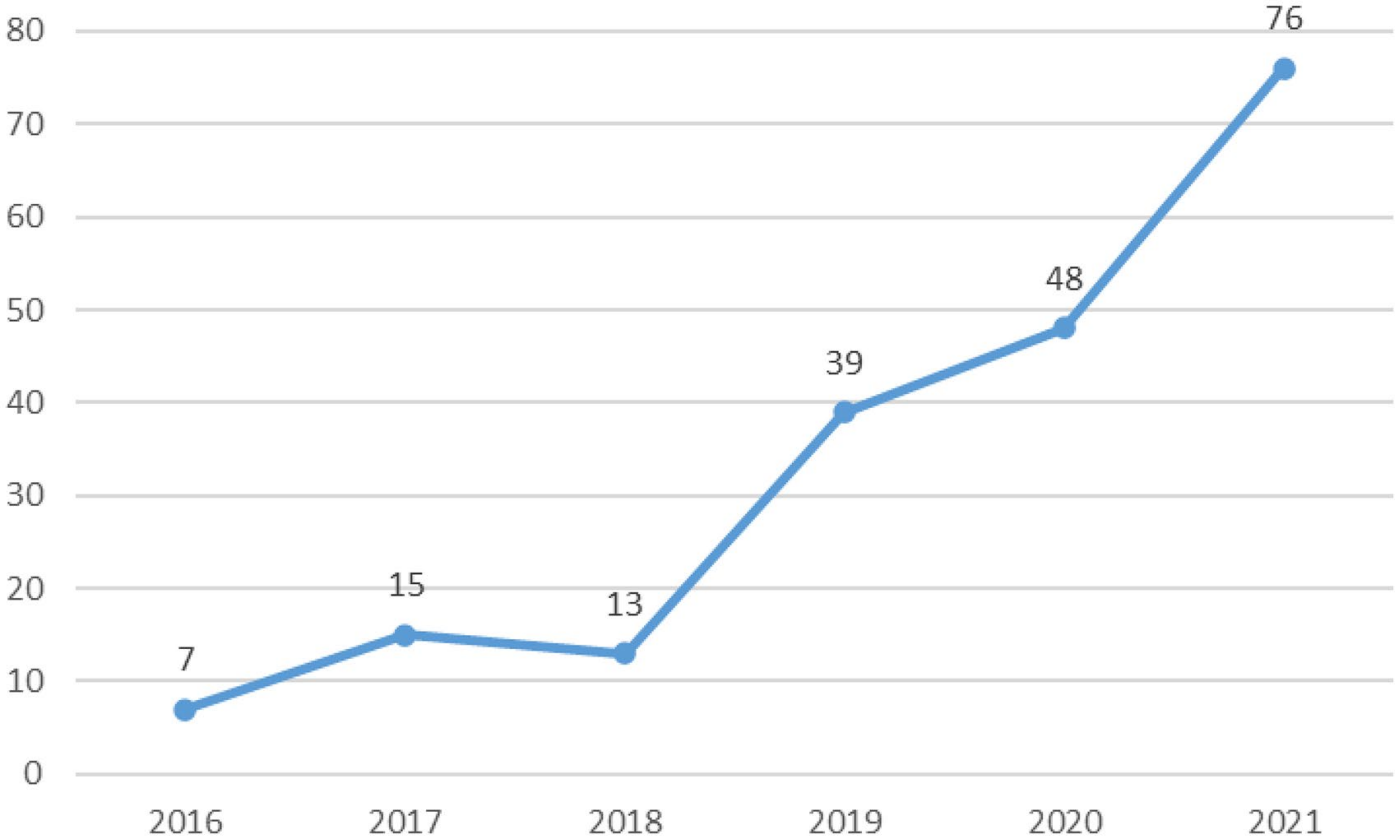
Annual publication 2016–2021.

The main retrieval time of this study was selected between 2016 and 2021. The article type is limited to papers, and finally, there are a total of 198 papers that fit the theme. The number of publications per year is shown in Figure 2. The number of articles published in a journal can reflect the research level and development level of the subject area, and the change in the popularity of a certain topic can be derived from it. Through the publication status from 2016 to 2021, it can be seen that there are more than seven papers published on this topic every year, and it can be seen that this field is at the beginning and continuous exploration stage. Through the summary of the number of published articles, it is found that the increase in the number of published articles after 2020 has increased rapidly, which may be closely related to the global new crown pneumonia epidemic, including special issues in 2021 such as “Sustainable Cross-Border Business Models,” which is aimed at cross-border businesses during the epidemic. Overall, since 2016, the research on cross border e-commerce has shown steady growth. In particular, by the end of 2021, the cumulative number of articles had reached 76. It can be seen that cross-border e-commerce has gradually become a hot topic and research frontier.

According to the 198 articles on cross-border e-commerce obtained from WOS data (see Table 1), the statistical description and analysis of the number of journal publications can help one observe the development of the disciplinary knowledge structure in the field of cross-border e-commerce and provide insights into subsequent research. The authors provide guidance for the submission of relevant papers. Among them, the Electronic Commerce Research journal has made the greatest contribution to cross-border e-commerce research. A total of 16 papers have been published, with a total of 99 citations and an average citation of 6.19. Among them, most of the literature uses the establishment of models to study the factors that affect consumers’ purchasing decisions and the relevant policies of customs on cross-border goods (Li and Li, 2019). Secondly, Sustainability is the second most published journal, with a cumulative citation rate of 90, but the average citation rate is higher than the second-ranked journal at 6.43. The third-ranked journal is the European Journal of Marketing, with eight publications and 15 cumulative citations, and an average citation rate of 1.88. The citation rate is lower than in the previous two journals. It is worth discussing the Journal of International Economic Law. This journal itself only includes five articles, but the cumulative citations amounted to 24, and the average citation rate reached 4.8, which is higher than the third-ranked journals. It mainly discusses the policy challenges of a data-driven economy and the impact of international tax and trade regimes on cross-border e-commerce (Mitchell and Mishra, 2019).

**Table 1:** Statistical table of journal publication.

Rank	Journals	Documents	TC	D|TC
1	Electronic Commerce Research	16	99	6.19
2	Sustainability	14	90	6.43
3	Journal of Coastal Research	8	15	1.88
4	Security and Communication Networks	7	1	0.14
5	Mobile Information Systems	6	7	1.17
6	Agro Food Industry Hi-Tech	5	7	1.4
7	Journal of International Economic Law	5	24	4.8
8	Journal of Korea Trade	5	14	2.8
9	Wireless Communications & Mobile Computing	5	2	0.4

TC, total citations; D|TC, average number of citations per article.

### Author’s cooperation network

**Figure 3:**
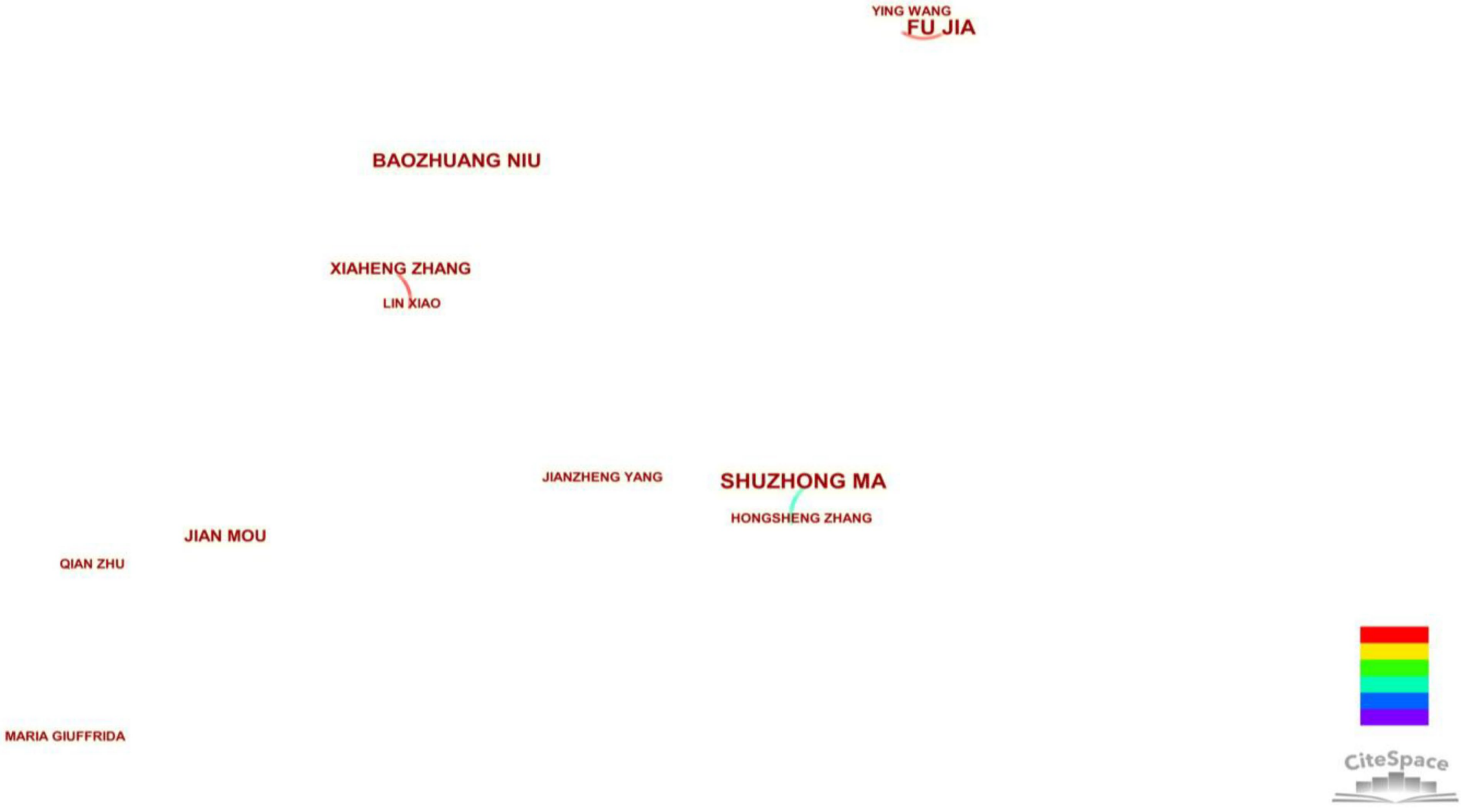
Author cooperation networks with more than three articles.

There are a total of 138 nodes and 91 connections in the author’s co-occurrence graph, and the network density is 0.0096. It can be seen that the cooperation network density between authors is low, the author’s cooperation relationship is not close enough, and the research authors are in a relatively scattered state. The five core authors in this study published 20 papers, accounting for 10.10% of all 198 papers, which also showed that the authors did not cooperate enough. The results of Figure 3 show that only three groups of authors form cooperation networks, namely, Fu Jia and Ying Wang, Lin Xiao and Xiaheng Zhang, and Shuzhong Ma and Hongsheng Zhang, which implies that though most CBEC topics attract the attention of a majority of researchers, few researchers conduct deep research on CBEC (at most five papers so far), and the cooperation among researchers is loose without a specific focus. Moreover, we can infer that, in terms of CBEC research, most researchers work individually or in small groups without established research authorities or centers. Nevertheless, despite the lack of specific cooperation networks, researchers follow some common research directions, such as how to improve service capabilities of supply chains, relevant strategic analysis of cross-border e-commerce, and analysis of customer purchase intentions.

The numbers of published papers per author are shown in Table 2. It can be seen that there are five authors who have published more than three papers. Among them, the highest number of publications was five times, accounting for 3.62% of the total number of scholars. The number of authors with a publishing frequency of 2 accounted for 4.35%, and the number of authors with a publishing frequency of 1 accounted for almost as high as 92.03%. Most of the researchers are dabbling in the field of “cross-border e-commerce” for the first time, and several of them have only published one study, which shows that there are fewer high-yield core authors who are deeply involved in the field of “cross-border e-commerce” and have more outstanding achievements. Although there are no scholars with a high publication output in this field, in terms of citation frequency, the article published by Li et al. (2018) has been cited 144 times, and it mainly studies how small and medium-sized enterprises enter into the cross-border e-commerce field on the Alibaba platform, so as to improve their market competitiveness. It can be seen that researchers pay more attention to this.

**Table 2:** Authors with more than three papers and their affiliated institutions.

Rank	Authors	Affiliation	Documents
1	Shuzhong Ma	University of Zhejiang	5
2	Jia Fu	University of Minjiang	5
3	Baozhuang Niu	South China University of Technology	4
4	Xiaheng Zhang	Nanchang Inst Sci & Technol	3
5	Barbara E Kahn	University of Xidian	3

When studying the citation rate of authors through the analysis of the WOS database, it is concluded that there are six articles with a high citation rate (more than 40) and two articles with a high citation rate, one of which is a study by Li et al. (2018). The study mainly investigates how entrepreneurs of under-capacity small and medium-sized enterprises (SMEs) can drive the digital transformation of their companies in the context of scarce resources and aims to expand the understanding of digital entrepreneurship and digital transformation through an inductive process model. Another highly cited article is that by Liu and Li (2020), which proposes a blockchain-based framework based on the cross-border e-commerce environment, integrates a series of blockchain-based models, and develops a corresponding set of techniques and methods to address product traceability issues. These techniques and methods contribute significant research value to integrating decentralized management systems in supply chains. Kim et al. (2017) probed into the influence of distance on CBEC and concluded that a shorter distance can raise the loyalty of buyers. Courier procedures were simplified to reduce buyers’ hesitation in purchase due to distance. Additionally, Valarezo et al. (2018) analyzed the CBEC data from Instituto Nacional de Estadística (INE) to identify factors influencing individuals’ purchases and deemed that males show a relatively higher acceptance of CBEC commodities. In addition, there are four other highly cited studies, and the main research topics are mostly regarding express logistics services and obstacles, advantages and disadvantages, and the driving factors of cross-border e-commerce (refer to Table 3).

**Table 3:** Most frequently cited authors.

Rank	Author (Year)	Title	Citations
1	Li et al. (2018)	Digital transformation by SME entrepreneurs: A capability perspective	144
2	Hsiao et al. (2017)	Logistics service design for cross-border E-commerce using Kansei engineering with text-mining-based online content analysis	76
3	Liu and Li (2020)	A blockchain-based framework of cross-border e-commerce supply chain	61
4	Kim et al. (2017)	Cross-Border Electronic Commerce: Distance Effects and Express Delivery in European Union Markets	57
5	Valarezo et al. (2018)	Drivers and barriers to cross-border e-commerce: Evidence from Spanish individual behavior	42
6	Deng and Wang (2016)	Early-mover advantages at cross-border business-to-business e-commerce portals	42

### Countries and institutions

**Figure 4:**
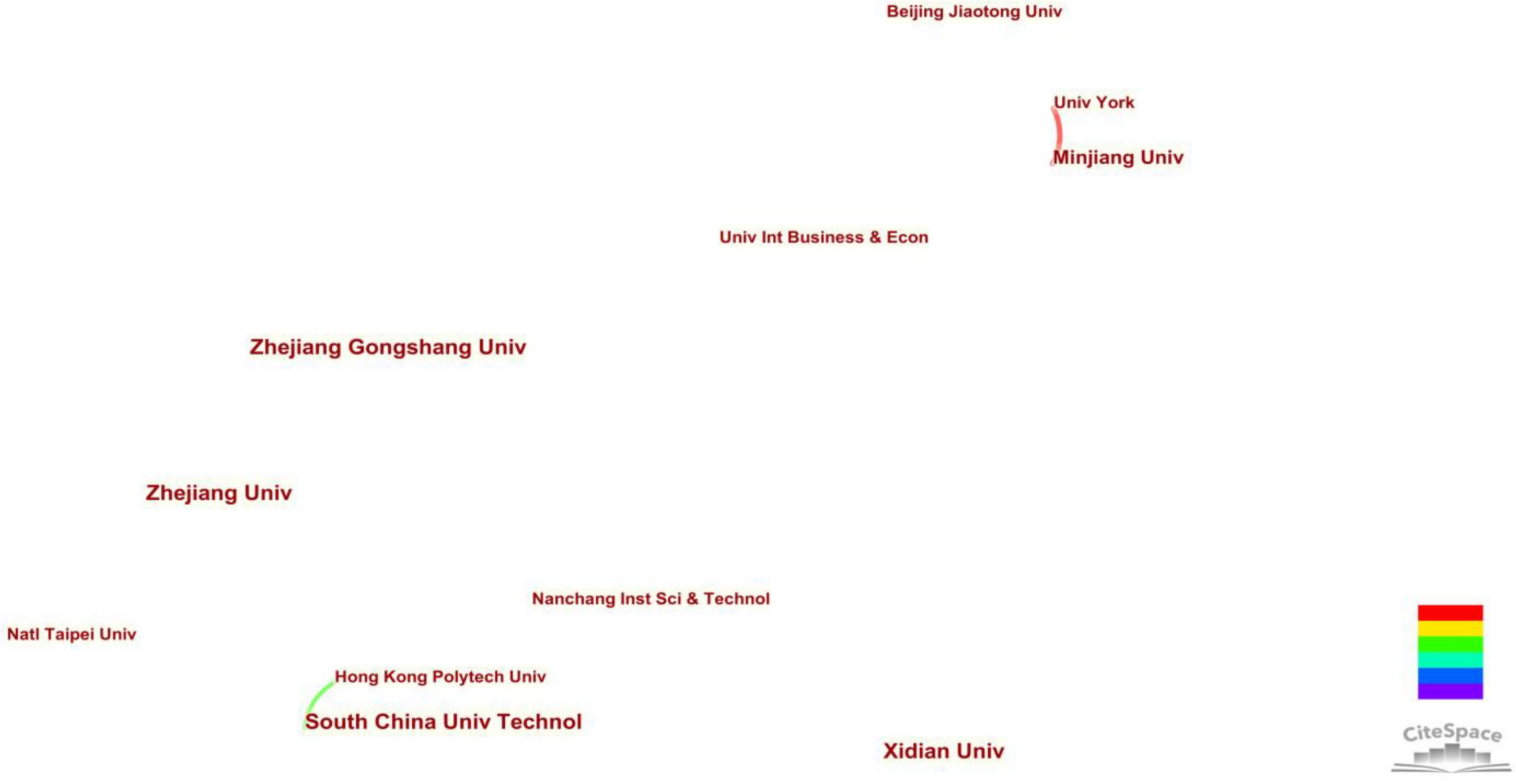
The organization’s cooperation network with more than three articles.

An analysis of the issuing institutions can reflect the high-yield research institutions and cooperation in this field. We draw the co-occurrence map of publishing institutions using CiteSpace, and obtain statistical information on the publishing situation of research institutions (See Table 4). The top research institutions in terms of published papers are Xidian University (five papers), Zhejiang Gongshang University (five papers), Zhejiang University (five papers), and other universities, indicating that these institutions conduct more in-depth cross-border e-commerce research and have high authority. From the perspective of the type of research institutions, the number of universities that have published more than three papers among 125 universities or research institutions has reached 13. It can be seen that the research on cross-border e-commerce is not carried out by a few universities, and it has gradually spread. From a geographical point of view, research institutions are mainly concentrated in China, the United Kingdom, the United States, and other countries, which are closely related to the current development of the country’s cross-border e-commerce field. The co-occurrence graph of the issuing agency has 124 nodes and 81 connections, and the network density is 0.0106 (See Figure 4). Most nodes are distributed in a sporadic state, and the connections between nodes are few and thin, indicating that the research institutions are scattered, the cooperative research results are few, and an academic research team that integrates and develops has not yet been formed. The existing cooperation between research institutions is mainly based on the close cooperation of several universities in China, and there is occasional cooperation between international institutions, such as the Hong Kong Polytechnic University, the South China University of Technology, Minjiang University, York University, and other universities that have mutual cooperation relationships.

**Table 4:** Organizations with more than three papers.

Ranking	Organizations	Documents
1	Xidian University	5
2	Zhejiang Gongshang University	5
3	Zhejiang University	5
4	South China University of Technology	5
5	Minjiang University	4
6	York University	3
7	Natl Taipei University	3
8	Hong Kong Polytechnic University	3
9	University of Int Business & Econ	3
10	Politecnico Milano University	3
11	Beijing Jiaotong University	3
12	Nanchang Inst Sci & Technol University	3

This study analyzes data recorded from 2016 to 2021 (See Table 5). A total of 24 countries have conducted relevant research in the field of “cross-border e-commerce,” and a total of five countries have published at least five articles. China is the country with the highest productivity in “cross-border e-commerce,” accounting for 63.96% (i.e., 126 publications are from Chinese authors). The United States is the country with the second highest output in the cross-border e-commerce sector, accounting for 6.09% (i.e., 12 publications are from authors in the United States). The United Kingdom is the third country in terms of output in the field of “cross-border e-commerce,” accounting for 5.08% (i.e., 10 publications are from United Kingdom authors) followed by South Korea (eight articles), and Taiwan (seven articles). Overall, China’s publication volume in the field of “cross-border e-commerce” exceeds that of the other four countries combined, which may be closely related to the development of China’s domestic cross-border e-commerce industry, which proves that China’s cross-border e-commerce industry leads the position in e-commerce.

**Table 5:** Countries with more than five publications.

Ranking	Countries	Documents
1	Peoples R China.	126
2	United States	12
3	England.	10
4	South Korea.	8
5	Taiwan.	7

### Keyword analysis

**Figure 5:**
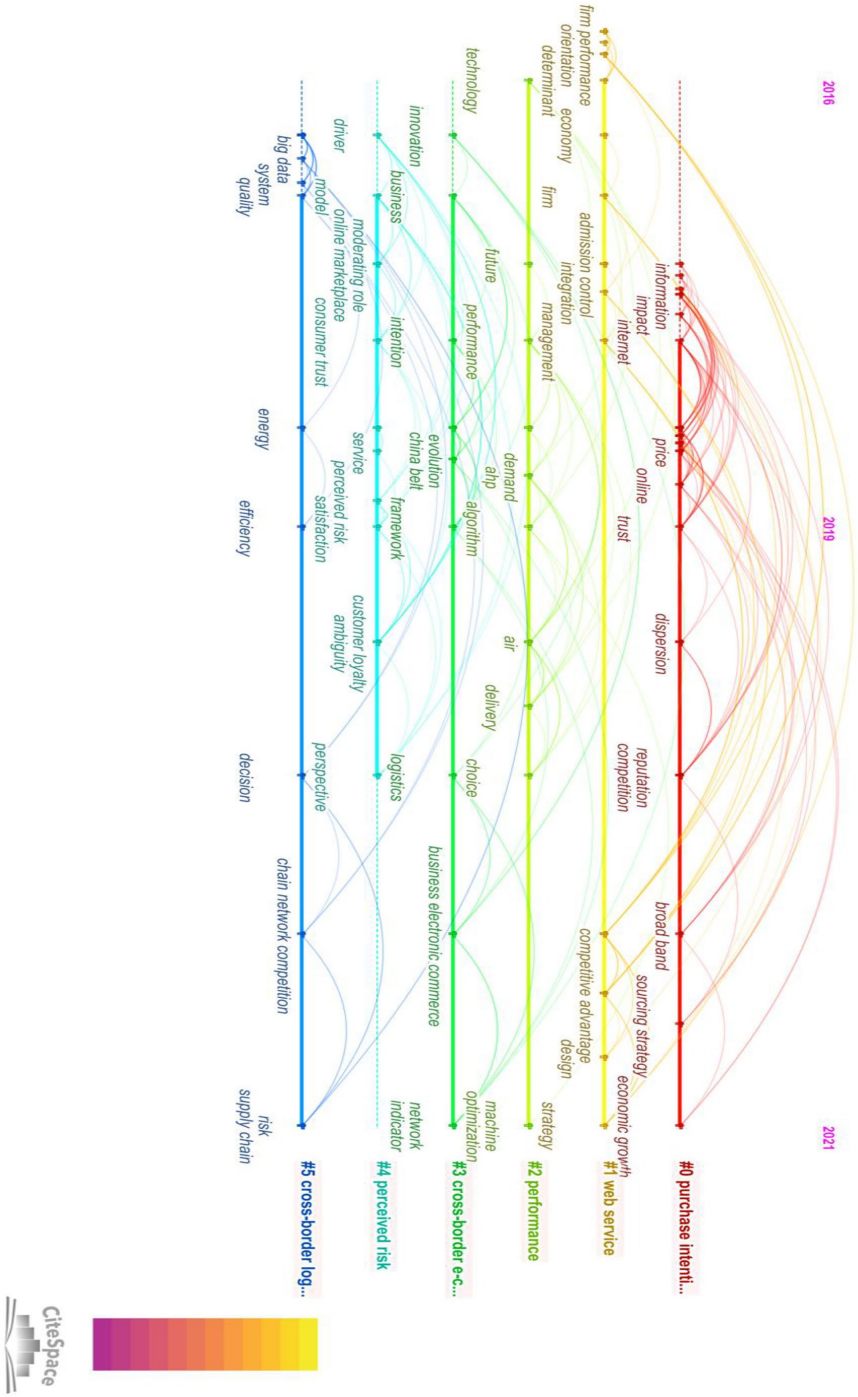
Timeline of the “Cross-border e-commerce” cited network.

The timeline graph provides a holistic view of the cluster time span and how those clusters are connected, with the results shown below. The keyword co-occurrence map can intuitively reflect the frequency of keywords in the research field, and the topic of the article can be clearly understood through keyword analysis. This study mainly formed six clusters. Nodes in each row represent keywords in each cluster, and links represent relationships between different keywords. Furthermore, the results between clusters show that the keyword correlations in each cluster are high. Cluster 0 is the largest cluster because it contains the most articles. Consecutive large nodes and extensive links in this cluster demonstrate its liveness, with the label of cluster 0 representing the most noteworthy topic among them. Clusters 0–1 also have larger nodes and involve more keywords, suggesting that they are relatively prominent topics in cross-border e-commerce. In the CiteSpace interface, we selected keyword as the node type, set the time slice to 1, used *g*-index for the selection criteria, and set *k* = 25. After running the software, the keyword co-occurrence map was obtained (Figure 5). There are 198 nodes and 620 connections in the graph, and the network density is 0.0562. Keywords with a frequency greater than or equal to 1 and a centrality greater than or equal to 0.1 are listed, as shown in the figure.

Cluster 0 is marked as “purchase intention,” which means that the study of this cluster can be generalized as the study of purchase intention. However, looking at the keywords of this category, words with strong relationships include trust, internet, impact, online, information, electronic commerce, b2c e-commerce, price, behavior, economic growth, eBay, channel, acceptance, word of mouth, sourcing strategy, social commerce, reputation, customer satisfaction, and competition. These words reflect that this topic mainly investigates the influence of various factors on customer purchase intention in cross-border e-commerce. For example, Guo et al. (2018) examined the impact of sellers’ trust on buyers and their perceived risk of chargeback fraud on sellers’ transaction buyer intentions in the context of cross-border e-commerce and developed a conceptual model that identified a set of institutional mechanisms to enhance seller trust and reduce their perceived risk. In addition, Chen J. and Yang L. (2021) explored the mediating effect of network structure embedded in customer experience and consumer purchase intention in the context of cross-border e-commerce. Tikhomirova et al. (2021) analyzed the interaction between consumers’ national culture, trust tendency, credibility perception, and other personal traits in different e-commerce environments and their impact on purchase intention.

Cluster 1 is labeled “web service,” which means that the study of this cluster can be summarized as the study of web services. However, looking at the keywords of this category, words with strong relationships include management, determinant, integration, strategy, design, firm, firm performance, orientation, decline, capability, organization, and competitive advantage. These words reflect that the issue is mainly in studying the operation and management of network services and supply chains and their important role in cross-border e-commerce. For example, Wang et al. (2020) elaborated on how cross-border e-commerce generates supply chain service capabilities, thereby improving the quality of supply chain relationships between e-commerce and other platform users. For another example, Fang (2021) starts with the concept and application principle of the green supply chain, analyzes how to integrate the green supply chain into each link of the logistics industry chain, and the problems existing in the operation of green logistics, and explores from the perspective of the green supply chain. The path of innovation and development of e-commerce enterprises and the method of introducing the concept of green environmental protection into the management decision-making of the logistics industry are proposed. In addition, Yu et al. (2021), based on the resource-based view and organizational capability theory, examined the impact of information technology (IT) on enterprise performance through supply chain integration (SCI) from the upstream and downstream perspectives of the supply chain.

Cluster 2 is labeled “performance,” which means that the study of this cluster can be generalized as the study of performance. However, looking at the keywords of this category, the words with strong relationships include technology, future, demand, algorithm, air, delivery, choice, machine, and optimization. These words reflect that the topic mainly explores various related performance capabilities and the impact of cross-border e-commerce development. For example, Ai et al. (2016) studied the impact of cross-border logistics performance on the development of the manufacturing industry. Through the comparison of various logistics performance factors, it was found that solving cross-border payment and regulatory legal issues can promote the development of cross-border e-commerce. In addition, Ma and Liang (2021) studied the factors affecting the export performance of cross-border e-commerce companies and found that, compared with industry competition, high-quality business services, diverse product choices, and low-priced products can promote the export performance of cross-border e-commerce enterprises. Furthermore, Xia and Liu (2021) adopted IoT tracking technology and multi-objective decision-making to propose an optimal management and coordination method to improve cross-border e-commerce supply chain performance. Through the sorting of the above cluster 2, the categories of performance mainly involve logistics performance, enterprise export performance, supply chain performance, and electronic trade performance.

Cluster 3 is labeled “cross-border e-commerce,” which means that the study of this cluster can be generalized as the study of cross-border e-commerce. Words with strong relationships include framework, business, innovation, network, indicator, evolution, China belt, and business electronic commerce. Comparing the citation frequencies of these words, logistics has the highest citation frequency, followed by intention, framework, and business. These words reflect the issue of exploring cross-border e-commerce. In the application of this clustering, consumers’ purchase intention is a hot topic of various researchers, which runs through the papers from 2016 to 2021. It can be seen that with the continuous change in the social environment, consumers’ purchase intentions will also change. Exploring the influencing factors of cross-border e-commerce consumers’ purchase intention can provide decision support for the management and operation of cross-border e-commerce, so as to better promote the development of cross-border e-commerce (Lu et al., 2021).

Cluster 4 is labeled “perceived risk,” which means that the study of this cluster can be generalized as the study of perceived risk. Looking at the keywords of this category, the words with strong relationships include customer loyalty, ambiguity, service, moderating role in the online marketplace, consumer trust, satisfaction, perspective, and drive. These keywords highlight how to influence customer satisfaction and purchase intention in the cross-border e-commerce environment. For example, Luo and Ye (2019) combined the characteristics of consumers on the IOO platform, with the structure of social capital. Starting from the dimensions of relationship and cognition, it draws the conclusion that consumers’ social capital of different dimensions can affect customer loyalty through different values. Others, such as Chen N. and Yang Y. P. (2021), believe that the cross-border e-commerce platform can be regarded as a social network, and the roles of platform companies, service providers, sellers, and consumers, among other, on the platform will be embedded in the network because of their mutual connection. Sellers can achieve the purpose of improving consumers’ purchase intentions by combining different strategies of network structure characteristics (network density, network centrality) and customer experience. Yang et al. (2021) combined perceived risk, transfer cost, and user loyalty VSL framework theory, constructed a new cross-border e-commerce mobile market user transfer intention framework, and analyzed user loyalty in the cross-border e-commerce mobile market, transfer costs, and transfer intentions.

Cluster 5 is labeled “cross-border logistics,” which means that the study of this cluster can be summarized as the study of cross-border logistics. Looking at the keywords of this category, the core words mainly include chain network competition, risk supply chain, decision, efficiency, energy, quality, system, and big data. In the application of clustering, researchers mainly focus on how to improve the efficiency of the supply chain and improve its ability to deal with risks (Liu and Li, 2020; Zhu and Zhou, 2020; Fang and Wang, 2021; Niu et al., 2021; Xia and Liu, 2021). In addition, following the trend of environmental protection, the research on green supply chains has gradually formed a trend. Fang (2021) starts with the concept and application principle of the green supply chain and analyzes how it can be integrated. The integration of supply chains into all aspects of the logistics industry chain, and the problems existing in the operation of green logistics, explore the path of innovation and development of e-commerce enterprises from the perspective of the green supply chain and put forward the method of introducing the concept of green environmental protection into the management and decision-making of the logistics industry (Zhang and Liu, 2021). Based on the action mechanism and model of the cross-border e-commerce green supply chain centered on customer behavior, and according to the green level evaluation requirements of the green supply chain, the analytic hierarchy process is used to evaluate the green supply chain.

## Discussion and implications

### Research discussion

This study mainly uses WOS for data analysis and finally selects 198 papers published between 2016 and 2021. The results of the data analysis draw the following conclusions:

1. Cross-border e-commerce research began to show an upward trend in 2019. In particular, in 2021, due to the impact of the new crown pneumonia epidemic, related cross-border e-commerce issues have attracted more attention from researchers.
2. On the issue of cross-border e-commerce, among the four major e-commerce journals in recent years, Electronic Commerce Research has shown steady growth in its number of publications. The main factor is that the journal was published in 2019 The Special Issue: Cross-border e-Commerce Initiatives under China’s Belt and Road Initiative and the Special Issue: Electronic Commerce in the Asia-Pacific region issued during 2020 all have related themes for submissions, so a large number of researchers have submitted papers to this paper. Contrarily, what is more interesting is that the open-source journal Sustainability ranks second in the number of publications on cross-border e-commerce issues.
3. With regard to highly cited literature, there are currently two highly cited articles in the WOS database, one of which is the only one with a cross-border e-commerce citation rate exceeding 100 (Li et al., 2018). Another study is an article published in the International Journal of Information Management in 2020, applying blockchain technology in the supply chain of cross-border e-commerce (Liu and Li, 2020), which was cited in a short 2-year period. The citation rate also broke 60, which also means that the application of artificial intelligence technology in cross-border e-commerce has become a hot spot in recent years.
4. For the part of the cooperation network between the country and the author, according to the findings of the data, the current research on the main hot spots of cross-border e-commerce is mainly in China, and there are cooperation networks in a few regions. However, it is still mainly based on cooperation with Chinese universities, such as Hong Kong Polytechnic University, South China University of Technology, Minjiang University, and York University, among others. This part also means that there is less international cooperation in cross-border e-commerce research.
5. The part of keyword network analysis mainly includes six clusters, namely purchase intention, web service, performance, cross-border e-commerce, perceived risk, and cross-border logistics. These six categories include the development of cross-border e-commerce website technology, the discussion of performance evaluation indicators for cross-border enterprises, cross-border transportation and logistics, and research on cross-border e-commerce consumer behavior. The topic of purchase intention has always been a research topic that cross-border e-commerce purchase intentions are keen on, but the topic of combining cross-border e-commerce supply chain and artificial intelligence is a topic that can be continuously paid attention to in the future.

### Implications for academic research

Based on the above research results, it can be inferred that research on cross-border e-commerce is still relatively nascent, especially because the related research is mainly based in Asia. Therefore, future research can discuss the differences in the current situation of cross-border e-commerce promotion in different countries or regions so as to further understand how to promote operations in different environments and regions. Further, most of the previous studies used questionnaires to analyze cross-border e-commerce consumer behavior. In the follow-up research, we should consider adopting the method of integrating neuroscience to analyze cross-border e-commerce purchase behavior. From the perspective of the topic, follow-up research can begin from artificial intelligence combined with cross-border e-commerce and cross-border e-commerce green supply chains, as outlined in previous studies by Zhu and Zhou (2020) and Fang and Wang (2021). Together with Liu and Li (2020) and other researchers, they discuss green supply chains. This direction is also in line with current mainstream issues such as carbon neutrality and carbon emissions. Artificial intelligence combined with cross-border e-commerce is also a direction that can be further discussed in the future (Xia and Liu, 2021).

### Implications for practical

In short, we have found that CBEC research presents different evolution and development paths. The research perspectives in recent years have evolved from consumers’ purchase behaviors, to CBEC development and policies. During the later period, researchers began studying the opportunities and challenges of CBEC. Also, the COVID-19 pandemic has accelerated the development of global CBEC topics. CBEC is ushering in a new round of opportunities and challenges. In conclusion, CBEC research has evolved from consumers’ purchase behaviors to the influences of new post-pandemic technologies and improvements in the global supply chain on CBEC.

### Research limitations

This study mainly uses the method of bibliometrics to analyze the literature, and there are certain limitations to this method. Regarding the method of data collection, this research mainly uses the WOS database to analyze the current situation of the literature on cross-border e-commerce published in SSCI/SCI from 2016 to 2021. Because the data analysis only covers articles included in SSCI/SCI, papers from conferences such as SCOUP or core workshops in the field of e-commerce (e.g., WHICEB, ICE-B) are not considered. Therefore, it is suggested that follow-up research should deepen the discussion on articles that address cross-border e-commerce topics included in core international conferences or databases.

## Data availability statement

The original contributions presented in the study are included in the article/supplementary material, further inquiries can be directed to the corresponding authors.

## Author contributions

YC and GLC designed the research and provided guidance throughout the entire research process. YC, GLC, and JS collected the references, did the literature analysis, and wrote the manuscript. ML, XM, and SW helped translating and offered modification suggestions. SW participated in the collecting, analyzing, and organizing of the literature. All authors contributed to the article and approved the submitted version.

## Conflict of interest

The authors declare that the research was conducted in the absence of any commercial or financial relationships that could be construed as a potential conflict of interest.

## Publisher’s note

All claims expressed in this article are solely those of the authors and do not necessarily represent those of their affiliated organizations, or those of the publisher, the editors and the reviewers. Any product that may be evaluated in this article, or claim that may be made by its manufacturer, is not guaranteed or endorsed by the publisher.

## References

[ref0001] Ai W. Yang J. Wang L. (2016). Revelation of cross-border logistics performance for the manufacturing industry development. Int. J. Mob. Commun. 14, 593–609. doi: 10.1504/IJMC.2016.079302

[ref12] Chen J. Yang L. (2021). A bibliometric review of volatility spillovers in financial markets: knowledge bases and research fronts. Emerg. Mark. Financ. Trade 57, 1358–1379. doi: 10.1080/1540496X.2019.1695119

[ref13] Chen N. Yang Y. P. (2021). The impact of customer experience on consumer purchase intention in cross-border E-commerce-Taking network structural embeddedness as mediator variable. J. Retail. Consum. Serv. 59:102344. doi: 10.1016/j.jretconser.2020.102344

[ref14] Cheng X. Su L. Zarifis A. (2019). Designing a talents training model for cross-border e-commerce: a mixed approach of problem-based learning with social media. Electron. Commer. Res. 19, 801–822. doi: 10.1007/s10660-019-09341-y

[ref15] Cho H. Lee J. (2017). Searching for logistics and regulatory determinants affecting overseas direct purchase: an empirical cross-national study. Asian J. Ship. Logist. 33, 11–18. doi: 10.1016/j.ajsl.2017.03.002

[ref20] Deng Z. Wang Z. (2016). Early-mover advantages at cross-border business-to-business e-commerce portals. J. Bus. Res. 69, 6002–6011. doi: 10.1016/j.jbusres.2016.05.015

[ref21] Ding F. Huo J. Campos J. K. (2017). The development of cross border E-commerce. Adv. Econ. Bus. Manag. Res. 37, 370–383. doi: 10.2991/ictim-17.2017.37

[ref24] Fang Z. Y. (2021). Innovative development of cross-border e-commerce based on green supply chain management. J. Environ. Prot. Ecol. 22, 2718–2726.

[ref25] Fang Z. Q. Wang Q. F. (2021). Cross-border E-commerce supply chain risk evaluation with fuzzy-ISM model. Secur. Commun. Net. 2021, 1–14. doi: 10.1155/2021/5155524

[ref27] Forrester Research (2019) Forrester Analytics: Online Cross-Border Retail Forecast, 2018 to 2023 (Global). Cambridge: Forrester Research Inc.

[ref28] Gao (2021). Study on the Intention of Foreign Trade Driven by Cross-Border E-Commerce Based on Blockchain Technology.

[ref29] Giuffrida M. Mangiaracina R. Perego A. Tumino A. (2020). Cross-border B2C e-commerce to China: an evaluation of different logistics solutions under uncertainty. Int. J. Phys. Distrib. Logist. Manag. 50, 355–378. doi: 10.1108/IJPDLM-08-2018-0311

[ref31] Guo Y. Bao Y. Stuart B. J. le-Nguyen K. (2018). To sell or not to sell: Exploring sellers' trust and risk of chargeback fraud in cross-border electronic commerce. Inf. Syst. J. 28, 359–383. doi: 10.1111/isj.12144

[ref32] Hsiao Y. H. Chen M. C. Liao W. C. (2017). Logistics service design for cross-border E-commerce using Kansei engineering with text-mining-based online content analysis. Telematics Inform. 34, 284–302. doi: 10.1016/j.tele.2016.08.002

[ref34] Huang W. L. Hu P. Tsai S. Chen X. D. (2021). The business analysis on the home-bias of E-commerce consumer behavior. Electron. Commer. Res. 21, 855–879. doi: 10.1007/s10660-020-09431-2

[ref35] Jia K. Wang P. Li Y. Chen Z. Jiang X. Lin C. L.. (2022). Research landscape of artificial intelligence and e-learning: a Bibliometric research. Front. Psychol. 13:795039. doi: 10.3389/fpsyg.2022.795039, PMID: 35250730 PMC8889112

[ref36] Kawa A. Zdrenka W. (2016). Conception of integrator in cross border ecommerce. Sci. J. Logist. 12:63. doi: 10.17270/J.LOG.2016.1.6

[ref37] Kim T. Y. Dekker R. Heij C. (2017). Cross-border electronic commerce: distance effects and express delivery in European Union markets. Int. J. Electron. Commer. 21, 184–218. doi: 10.1080/10864415.2016.1234283

[ref45] Li C. Chan T. (2016). “Ten China commercial highlights 2016 series: cross-border e-commerce import businesses see rapid growth; new channels for commercial enterprises to transform and innovate.” Available at: www.fbicgroup.com/sites/default/files/Ten%20Highlights%20of%20/China’s%20Commercial%20Sector%202016_07.pdf (Accessed May 27, 2019).

[ref46] Li G. Li N. (2019). Customs classification for cross-border e-commerce based on text-image adaptive convolutional neural network. Electron. Commer. Res. 19, 779–800. doi: 10.1007/s10660-019-09334-x

[ref47] Li L. Su F. Zhang W. Mao J. Y. (2018). Digital transformation by SME entrepreneurs: a capability perspective. Inf. Syst. J. 28, 1129–1157. doi: 10.1111/isj.12153

[ref0002] Lin C. L. Chen Z. Jiang X. Chen G. L. Jin P. (2022). Roles and research trends of neuroscience on major information systems journal: a bibliometric and content analysis. Front. Neurosci. 16. doi: 10.3389/fnins.2022.872532 PMC9382099 35992932

[ref003] Liu C. L. Lai P. Y. (2016). Impact of external integration capabilities of third-party logistics providers on their financial performance. Int. J. Logist. Manag. 27, 263–283. doi: 10.1108/IJLM-09-2014-0155

[ref48] Liu Z. Li Z. (2020). A blockchain-based framework of cross-border e-commerce supply chain. Int. J. Inf. Manag. 52:102059. doi: 10.1016/j.ijinfomgt.2019.102059

[ref004] Lu C. W. Lin G. H. Wu T. J. Hu I. Chang Y. C. (2021). Influencing factors of cross-border e-commerce consumer purchase intention based on wireless network and machine learning. Secur. Commun. Netw. 2021. doi: 10.1155/2021/9984213

[ref001] Luo Y. M. Ye Q. W. (2019). Understanding consumers’ loyalty to an online outshopping platform: the role of social capital and perceived value. Sustainability 11:18.

[ref50] Ma S. Z. Liang Q. H. (2021). Industry competition, life cycle and export performance of China's cross-border e-commerce enterprises. Int. J. Technol. Manag. 87, 171–204. doi: 10.1504/IJTM.2021.120926

[ref52] Mitchell A. D. Mishra N. (2019). Regulating cross-border data flows in a data-driven world: how WTO law can contribute. J. Int. Econ. Law 22, 389–416. doi: 10.1093/jiel/jgz016

[ref54] Mou J. Cui Y. Kurcz K. (2019a). Bibliometric and visualized analysis of research on major e-commerce journals using Citespace. J. Electron. Commer. Res. 20, 219–237.

[ref55] Mou J. Ren G. Qin C. Kurcz K. (2019b). Understanding the topics of export cross-border e-commerce consumers feedback: an LDA approach. Electron. Commer. Res. 19, 749–777. doi: 10.1007/s10660-019-09338-7

[ref56] Mou J. Shin D. H. Cohen J. (2016). Health beliefs and the valence framework in health information seeking behaviors. Inf. Technol. People 29, 876–900. doi: 10.1108/ITP-06-2015-0140

[ref005] Nagariya R. Kumar D. Kumar I. (2021). Service supply chain: from bibliometric analysis to content analysis, current research trends and future research directions. Benchmarking 28, 333–369.

[ref59] Niu B. Z. Chen K. Chen L. Ding C. Yue X. (2021). Strategic waiting for disruption forecasts in cross-border e-commerce operations. Prod. Oper. Manag. 30, 2840–2857. doi: 10.1111/poms.13371

[ref67] Sinkovics R. R. Yamin M. Hossinger M. (2007). Cultural adaptation in cross border e-commerce: a study of German companies. J. Electron. Commer. Res. 8, 221–235.

[ref69] Strzelecki A. (2019). Key features of e-tailer shops in adaptation to cross-border e-commerce in the EU. Sustain. For. 11:1589. doi: 10.3390/su11061589

[ref70] Su Y.-S. Lin C.-L. Chen S.-Y. Lai C.-F. (2020). Bibliometric study of social network analysis literature. Lib. Hi Tech 38, 420–433. doi: 10.1108/lht-01-2019-0028

[ref006] Tikhomirova A. Huang J. Chuanmin S. Khayyam M. Ali H. Khramchenko D. S. (2021). How Culture and Trustworthiness Interact in Different E-Commerce Contexts: A Comparative Analysis of Consumers’ Intention to Purchase on Platforms of Different Origins. Front. Psychol. 12:746467. doi: 10.3389/fpsyg.2021.746467 34675852 PMC8523785

[ref75] UNCTAD (2016). CROSS-BORDER E-COMMERCE TRADE DATA UNCTAD technical notes on ICT for development [WWW document]. (Accessed January 2, 2018).

[ref77] Valarezo Á. Pérez-Amaral T. Garín-Muñoz T. García I. H. López R. (2018). Drivers and barriers to cross-border e-commerce: evidence from Spanish individual behavior. Telecommun. Policy 42, 464–473. doi: 10.1016/j.telpol.2018.03.006

[ref007] Wang Y. Jia F. Schoenherr T. Gong Y. Chen L. J. (2020). Cross-border e-commerce firms as supply chain integrators: The management of three flows. Ind. Mark. Manag. 89, 72–88. doi: 10.1016/j.indmarman.2019.09.004

[ref81] Wei K. Li Y. Zha Y. Ma J. (2019). Trust, risk and transaction intention in consumer-to-consumer e-marketplaces: An empirical comparison between buyers’ and sellers’ perspectives. Ind. Manag. Data Syst. 119, 331–350. doi: 10.1108/IMDS-10-2017-0489

[ref83] Wu L. Y. Chen K. Y. Chen P. Y. Cheng S. L. (2014). Perceived value, transaction cost, and repurchase-intention in online shopping: a relational exchange perspective. J. Bus. Res. 67, 2768–2776. doi: 10.1016/j.jbusres.2012.09.007

[ref86] Xia L. Liu S. T. (2021). Intelligent IoT-based cross-border e-commerce supply chain performance optimization. Wirel. Commun. Mob. Comput. 2021, 1–13. doi: 10.1155/2021/996192535573891

[ref87] Xiao L. Guo F. Yu F. Liu S. (2019). The effects of online shopping context cues on consumers’ purchase intention for cross-border E-commerce sustainability. Sustain. For. 11:2777. doi: 10.3390/su11102777

[ref88] Xu H. Q. Chung C. C. Yu C. (2022). Visualizing research trends on culture neuroscience (2008-2021): a Bibliometric analysis. Front. Psychol. 13:884929. doi: 10.3389/fpsyg.2022.884929, PMID: 35602732 PMC9121129

[ref008] Yang G. J. Wang Y. J. Lu F. T. Yu L. H. Ma S. Z. (2021). What Determines the Pattern of China’s Cross-Border E-Commence With the World? J. Glob. Inf. Manag. 29, 55–70. doi: 10.4018/JGIM.20210901.oa4

[ref92] Yu Y. B. Huo B. Zhang Z. (. J.). (2021). Impact of information technology on supply chain integration and company performance: evidence from cross-border e-commerce companies in China. J. Enterp. Inf. Manag. 34, 460–489. doi: 10.1108/JEIM-03-2020-0101

[ref002] Zhang X. H. Liu S. K. (2021). Action mechanism and model of cross-border e-commerce green supply chain based on customer behavior. Math. Probl. Eng. 2021:11.

[ref009] Zhang J. L. Wu T. Fan Z. P. (2019). Research on Precision Marketing Model of Tourism Industry Based on User’s Mobile Behavior Trajectory. Mob. Inf. Syst. 2019:14. doi: 10.1155/2019/6560848

[ref010] Zhu Z. Jin Y. Su Y. Jia K. Lin C. L. Liu X. (2022). Bibliometric-Based Evaluation of the Neuromarketing Research Trend: 2010–2021. Front. Psychol. 13:872468. doi: 10.3389/fpsyg.2022.872468 35983212 PMC9380815

[ref94] Zhu Q. Zhou H. (2020). the fractal statistical model of transregional and transnational e-commerce enterprises supply chain sequence. Fractals 28:2040022. doi: 10.1142/S0218348X20400228

